# Simplifying ANCA-associated vasculitis classification with ANCA specificity: a retrospective analysis

**DOI:** 10.1007/s10067-025-07397-w

**Published:** 2025-03-18

**Authors:** Lina Zhang, Jing Zhang, Jing Xu, Qian Guo, Yadan Zou, Xuewu Zhang, Kuanting Wang, Lianjie Shi, Shengguang Li

**Affiliations:** 1 Department of Rheumatology and Immunology, Peking University International Hospital, 102206 Beijing, China; 2 Department of Rheumatology and Immunology, Peking University People’s Hospital, Beijing, China; 3 Department of Rheumatology and Immunology, Peking University Shougang Hospital, Beijing, China

**Keywords:** ANCA-associated vasculitis (AAV), ANCA specificity, Clinical utility, Diagnostic classification, EMA algorithm, Kappa statistics, MPO-ANCA, PR3-ANCA, 2022 ACR/EULAR criteria

## Abstract

**Objective:**

This study aimed to evaluate the utility of ANCA specificity as a primary criterion for classifying AAV subtypes to simplify the diagnostic process without compromising accuracy.

**Methods:**

A retrospective cohort study was conducted involving 310 patients diagnosed with AAV between January 2015 and December 2023 across three tertiary care centers affiliated with Peking University. Patients were reclassified using three methods: the European Medicines Agency (EMA) algorithm, the 2022 American College of Rheumatology/European Alliance of Associations for Rheumatology (ACR/EULAR) criteria, and ANCA specificity-based classification. Concordance between classification systems was assessed using Cohen’s kappa coefficients.

**Results:**

ANCA specificity-based classification demonstrated substantial to almost perfect agreement with the 2022 ACR/EULAR criteria for MPA/MPO-AAV (kappa = 0.806) and GPA/PR3-AAV (kappa = 0.663). Many patients initially classified as GPA under the EMA algorithm were reclassified as MPA when using ANCA specificity. EGPA classification remained consistent across all methods (kappa = 0.725 between EMA and ACR/EULAR), suggesting that ANCA specificity is less critical for EGPA. The use of ANCA specificity simplified the classification process, aligning closely with the underlying pathophysiology of AAV subtypes.

**Conclusion:**

ANCA specificity serves as a valuable adjunct in the classification of AAV, particularly for distinguishing between MPA and GPA. Utilizing ANCA serotypes can simplify the diagnostic process, potentially facilitating earlier diagnosis and targeted treatment. For EGPA, traditional classification criteria remain effective. Incorporating ANCA specificity into clinical practice may enhance diagnostic accuracy and improve patient outcomes in AAV management.

**Table:** 

**Key Points** *• ANCA-based classification aligns strongly with the 2022 ACR/EULAR criteria for MPA and GPA, providing a simplified diagnostic approach.* *• Adopting this approach can streamline the classification process, reduce invasive procedures, and enable earlier diagnosis while maintaining high concordance with established systems.*

## Introduction

Antineutrophil cytoplasmic antibody-associated vasculitis (AAV) encompasses a group of systemic autoimmune disorders characterized by inflammation of small to medium-sized blood vessels, leading to organ damage and significant morbidity. The primary subtypes—granulomatosis with polyangiitis (GPA), microscopic polyangiitis (MPA), and eosinophilic granulomatosis with polyangiitis (EGPA)—often present with overlapping clinical and histopathological features, complicating accurate diagnosis and timely therapeutic intervention [1].

Current classification systems, such as the European Medicines Agency (EMA) algorithm and the recently updated 2022 American College of Rheumatology/European Alliance of Associations for Rheumatology (ACR/EULAR) criteria, integrate a combination of clinical manifestations, histopathological findings, and serological markers [2–5]. While these comprehensive frameworks enhance diagnostic specificity, their complexity can delay diagnosis and initiation of appropriate treatment, potentially adversely affecting patient outcomes.

Advancements in immunoserological testing have highlighted the significance of antineutrophil cytoplasmic antibodies (ANCAs), particularly those targeting myeloperoxidase (MPO) and proteinase 3 (PR3), in the pathogenesis and diagnosis of AAV [6]. MPO-ANCA is predominantly associated with MPA, whereas PR3-ANCA is more frequently observed in GPA [7]. This serological distinction aligns with differing clinical phenotypes and prognoses, suggesting that ANCA specificity could serve as a valuable tool for simplifying AAV classification while maintaining diagnostic accuracy [8].

Several studies have proposed utilizing ANCA specificity as a primary criterion for classifying AAV subtypes [9–11]. Such an approach could streamline the diagnostic process, reduce reliance on invasive procedures like biopsies, and facilitate earlier initiation of targeted therapies. However, the applicability and reliability of ANCA-based classification in routine clinical practice require further validation, particularly concerning its concordance with established classification systems.

In contrast, EGPA often presents with asthma and eosinophilia, and its classification appears less dependent on ANCA status [12]. This suggests that while ANCA specificity may enhance the classification of GPA and MPA, a different approach might be necessary for EGPA to ensure comprehensive patient assessment.

Given these considerations, our study aims to evaluate the feasibility and clinical utility of using ANCA specificity as a primary criterion for classifying AAV subtypes. By retrospectively analyzing data from 310 patients diagnosed with AAV in three tertiary care centers affiliated with Peking University, we seek to determine whether ANCA-based classification aligns with the 2022 ACR/EULAR criteria and whether it can effectively simplify the diagnostic process without compromising accuracy. We hypothesize that ANCA specificity will show substantial agreement with established classification systems for GPA and MPA, thereby supporting its integration into clinical practice to improve patient outcomes.

## Patients and methods

### Study design and setting

This retrospective cohort study was conducted at three tertiary care centers affiliated with Peking University, leveraging a comprehensive database of vasculitis patients with standardized ANCA testing and classification protocols. The study period spanned from January 2015 to December 2023, encompassing patients diagnosed with ANCA-associated vasculitis (AAV) based on clinical, histopathological, and serological criteria.

### Participants

We screened 313 patients with suspected AAV and ultimately included 310 who met the final criteria. All included individuals had clinical, serological, and/or histopathological data, satisfying the 2022 ACR/EULAR criteria for MPA, GPA, or EGPA [3–5]. Patients with drug-induced vasculitis, incomplete data, or other forms of vasculitis (e.g., polyarteritis nodosa) were excluded.

### Data collection

Clinical data were retrospectively extracted from the electronic health records using a standardized data collection form. The collected variables included:

- (1) Demographics: age at diagnosis and sex.
- (2) Clinical features: symptoms at presentation and organ involvement.
- (3) Laboratory findings: ANCA testing results (MPO-ANCA, PR3-ANCA), eosinophil counts, and renal function tests.
- (4) Histopathology: biopsy results when available.
- (5) Disease activity assessment: Disease activity was evaluated using the Birmingham Vasculitis Activity Score (BVAS-1994), a validated tool that quantifies disease severity based on organ involvement, with higher scores indicating more active disease [13].

ANCA testing was performed using indirect immunofluorescence (IIF) and enzyme-linked immunosorbent assay (ELISA) methods adhering to standardized protocols.

### Classification procedures

Each patient was reclassified using three different methods:

1. EMA algorithm: Patients were classified according to the European Medicines Agency algorithm, which incorporates clinical manifestations, histopathological findings, and ANCA status [2].
2. 2022 ACR/EULAR criteria: patients were evaluated against the 2022 ACR/EULAR classification criteria for AAV, which provide specific point-based scores for GPA, MPA, and EGPA based on clinical, laboratory, and imaging findings [3–5].
3. ANCA specificity-based classification: Patients were classified primarily based on their ANCA serotype:
  o MPO-AAV (MPA): Patients positive for MPO-ANCA (or P-ANCA) were classified as having microscopic polyangiitis.
  o PR3-AAV (GPA): Patients positive for PR3-ANCA (or C-ANCA) were classified as having granulomatosis with polyangiitis.
  o ANCA-negative AAV: Patients negative for both MPO-ANCA and PR3-ANCA were classified based on clinical features, with emphasis on EGPA if eosinophilia and asthma were present.
  o Dual-Positive ANCA: Patients positive for both MPO-ANCA and PR3-ANCA were carefully evaluated and categorized based on predominant clinical features and organ involvement.

### Statistical analysis

Descriptive statistics were computed for demographic and clinical variables. Cohen’s kappa coefficients measured the degree of agreement among the classification systems. Kappa values of 0.61–0.80 indicated substantial agreement, while 0.81–1.00 signaled almost perfect agreement [14]. Statistical analyses were performed using IBM SPSS Statistics 26.0 (IBM Corp., Armonk, NY). A *p*-value of less than 0.05 was considered statistically significant.

### Ethical considerations

This study was conducted in accordance with the ethical standards of the institutional and national research committees and with the 1964 Helsinki Declaration and its later amendments. The study protocol was approved by local Institutional Review Boards and Ethics Committees. Given the retrospective nature of the study and the use of de-identified patient data, the need for informed consent was waived.

All patient data were anonymized and handled confidentially. Data collection and analysis complied with the Personal Information Protection Law (PIPL) of the People’s Republic of China, ensuring data privacy and security.

## Results

### Patient demographics

The study included a total of 310 patients diagnosed with antineutrophil cytoplasmic antibody-associated vasculitis (AAV). The cohort comprised 165 females (53.2%) and 145 males (46.8%), with a mean age at diagnosis of 60.7 years (interquartile range (IQR), 53.0 years) (Table 1). MPO-ANCA positivity was seen in 207 (66.8%) patients, PR3-ANCA in 55 (17.7%), and dual positivity in 16 (5.2%). Another 32 (10.3%) were ANCA-negative. The BVAS-1994 score averaged 18.2 ± 8.1, reflecting moderate to high disease activity (Table 1).

**Table 1:** Characteristics of AAV patients at diagnosis

Variables	Values
Total patients	310 (100%)
Age at diagnosis	60.7 years (IQR, 53.0)
Male sex, *n* (%)	145 (46.8%)
ANCA positivity	
MPO-ANCA (or P-ANCA) positivity	207 (66.8%)
PR3-ANCA (or C-ANCA) positivity	55 (17.7%)
Both ANCA positivity	16 (5.2%)
ANCA negativity	32 (10.3%)
BVAS-1994 score	18.2 ± 8.1
Symptoms at presentation	
General	193 (62.3%)
Arthritis/arthralgia	52 (16.8%)
Mucocutaneous	36 (11.6%)
Ocular Symptoms	24 (7.7%)
ENT/upper respiratory tract	124 (40.0%)
Pulmonary hemorrhage/interstitial lung disease	181 (58.4%)
Cardiovascular	36 (11.6%)
Gastrointestinal	87 (28.1%)
Renal involvement	166 (53.5%)
Central nervous system	27 (8.7%)
Peripheral nervous system	69 (22.3%)

*ANCA* antineutrophil cytoplasmic antibody, *BVAS* Birmingham vasculitis activity score, *ENT* ear nose and throat, *IQR* interquartile range, *MPO* myeloperoxidase, *PR3* proteinase 3

With respect to clinical manifestations, 193 patients (62.3%) presented with general symptoms, 181 (58.4%) had pulmonary involvement (hemorrhage and/or interstitial lung disease), 166 (53.5%) exhibited renal involvement, and 124 (40.0%) experienced ear, nose, and throat (ENT) symptoms. Additional systemic features are detailed in Table 1.

### Classification outcomes

#### EMA algorithm classification (Fig. 1)

**Fig. 1:**
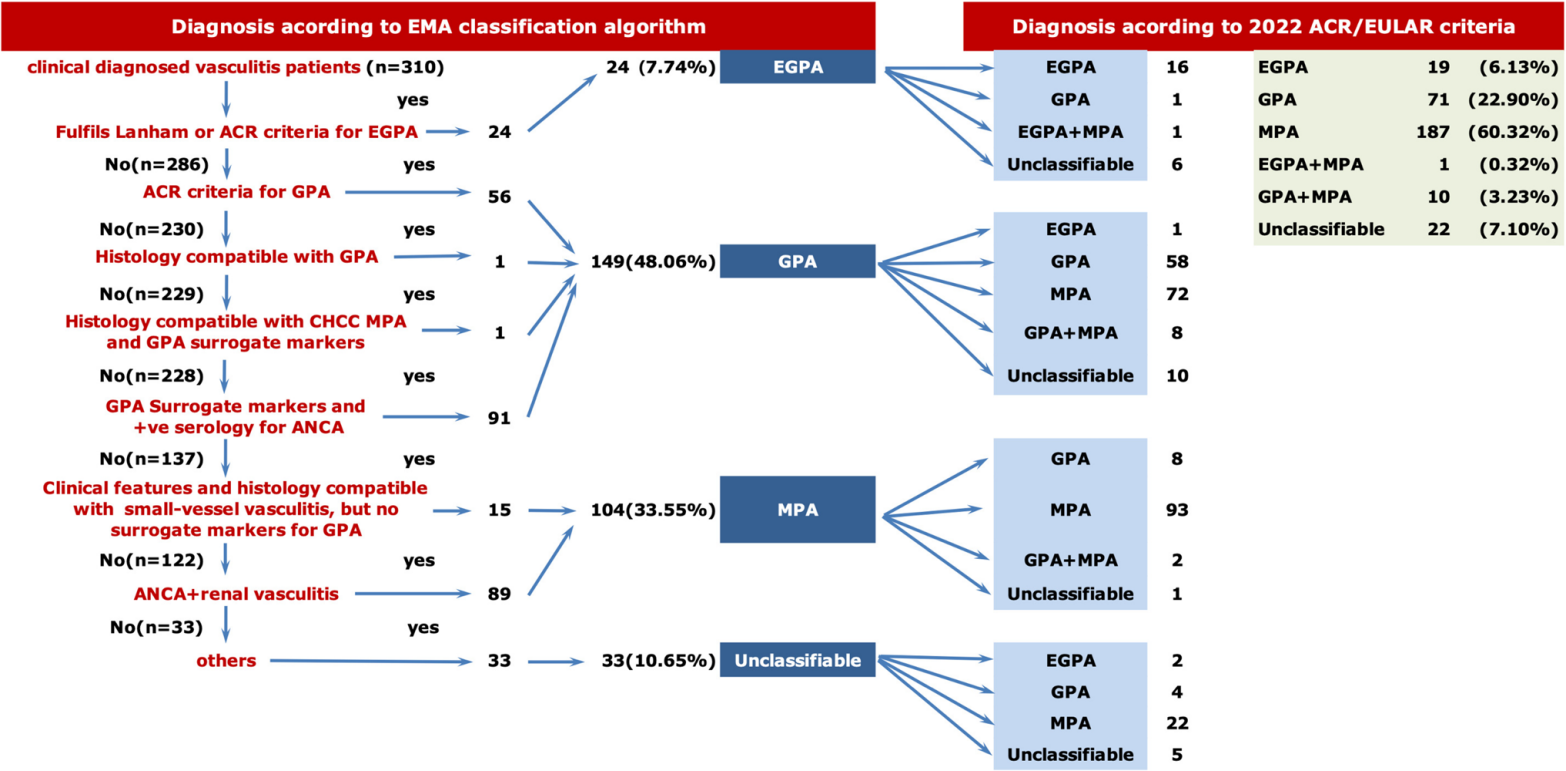
Comparison of classification results between the EMA algorithm and the 2022 ACR/EULAR criteria. The flow diagram illustrates the reclassification of patients from the EMA algorithm to the 2022 ACR/EULAR criteria, highlighting the significant shift of patients from GPA to MPA classification. MPA, microscopic polyangiitis; GPA, granulomatosis with polyangiitis; EGPA, eosinophilic GPA

Using the EMA algorithm, 149 patients (48.1%) were classified as GPA, 104 (33.5%) as MPA, 24 (7.7%) as EGPA, and 33 (10.6%) unclassified.

#### ACR/EULAR criteria classification (Fig. 1)

When applying the 2022 ACR/EULAR criteria, 187 patients (60.3%) met MPA criteria, 71 (22.9%) were labeled GPA, 19 (6.1%) were EGPA, 11 (3.5%) fulfilled more than one subtype, and 22 (7.1%) remained unclassified.

#### ANCA specificity-based classification

Based on the ANCA serotype:

- MPO-AAV (MPA): a total of 207 patients (66.8%)
- PR3-AAV (GPA): a total of 55 patients (17.7%)
- ANCA-Negative AAV/EGPA: a total of 32 patients (10.3%)
- Dual-Positive ANCA (Overlap): a total of 16 patients (5.2%)

### Classification transitions

#### EMA algorithm vs. 2022 ACR/EULAR criteria

**Fig. 2:**
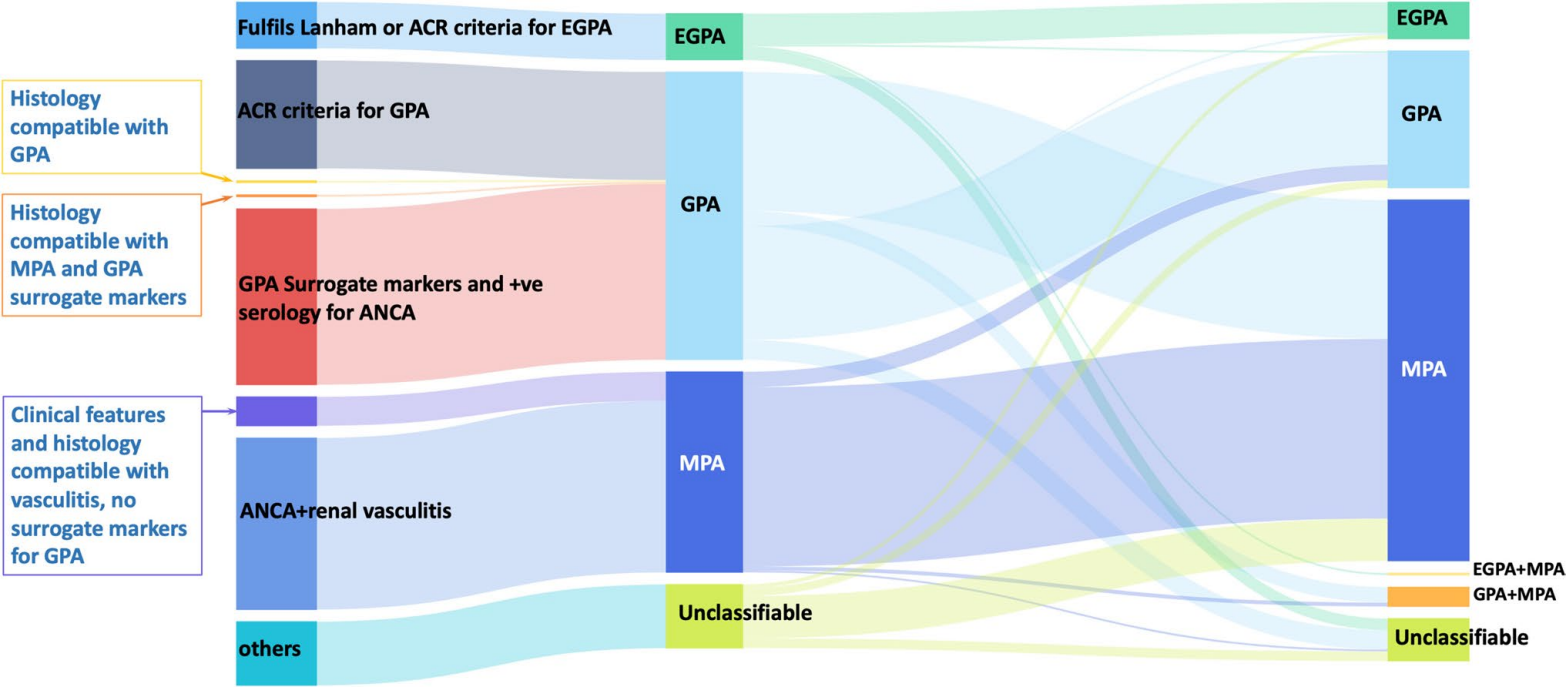
Sankey diagram showing transitions between the EMA algorithm and 2022 ACR/EULAR criteria. The diagram visualizes the patient distribution changes when reclassified based on the 2022 ACR/EULAR criteria, demonstrating the discrepancies between traditional classifications. ANCA, antineutrophil cytoplasmic antibody; MPA, microscopic polyangiitis; GPA, granulomatosis with polyangiitis; EGPA, eosinophilic GPA

A significant reclassification was observed when transitioning from the EMA algorithm to the 2022 ACR/EULAR criteria (Figs. 1 and 2). Notably:

- GPA to MPA shift: Out of the 149 patients initially classified as GPA by the EMA algorithm, 72 patients (48.3%) were reclassified as MPA under the 2022 ACR/EULAR criteria.
- MPA classification consistency: Most patients initially classified as MPA (104 patients) by the EMA algorithm remained classified as MPA (93 patients, 89.4%) under the 2022 ACR/EULAR criteria.
- EGPA classification stability: EGPA classifications were largely consistent between both systems.

#### EMA algorithm vs. ANCA specificity

**Fig. 3:**
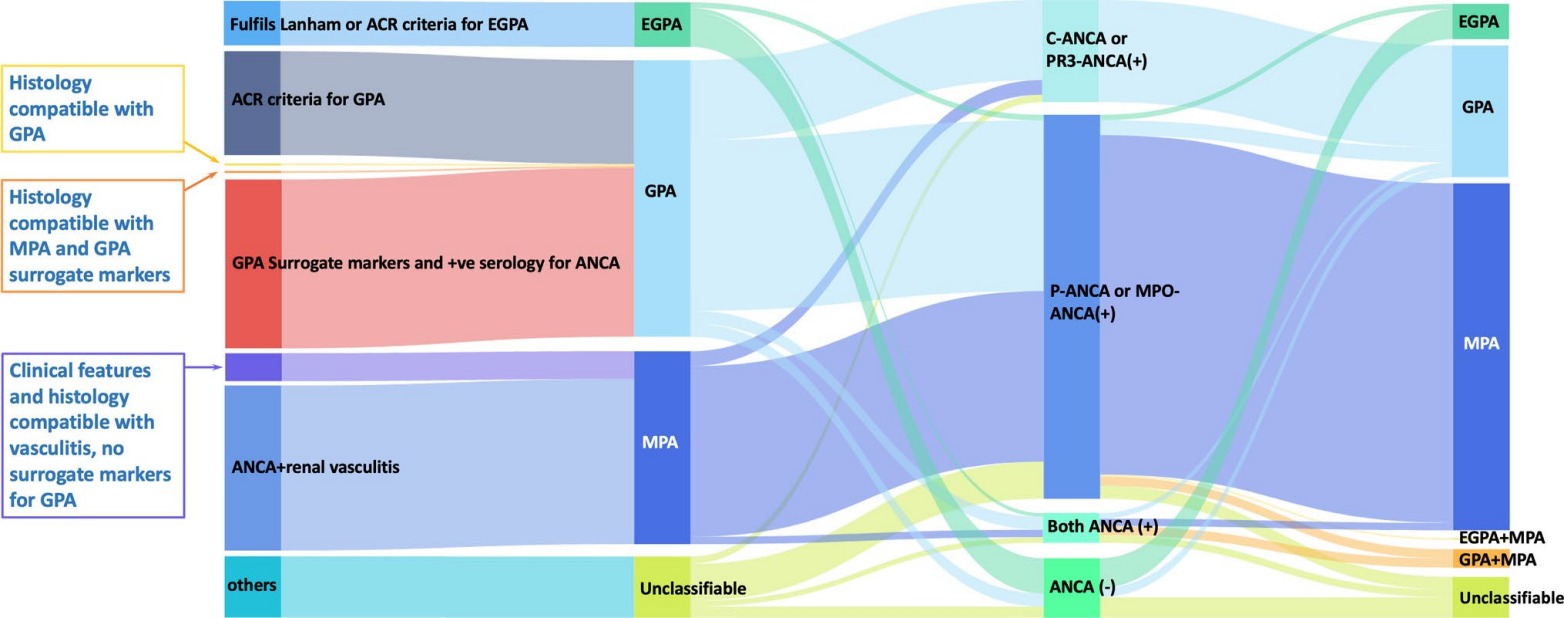
Sankey diagram illustrating classification transitions among the EMA algorithm, 2022 ACR/EULAR criteria, and ANCA specificity. This comprehensive diagram shows the multi-dimensional classification transitions, emphasizing the alignment between ANCA specificity and the 2022 ACR/EULAR criteria. AAV, ANCA-associated vasculitis; ANCA, antineutrophil cytoplasmic antibody; MPA, microscopic polyangiitis; GPA, granulomatosis with polyangiitis; EGPA, eosinophilic GPA; MPO, myeloperoxidase; P, perinuclear; PR3, proteinase 3; C, cytoplasmic

Comparing the EMA algorithm with ANCA specificity-based classification (Fig. 3):

- GPA (EMA) and PR3-AAV concordance: Only 42 out of 149 patients (28.2%) classified as GPA by the EMA algorithm were PR3-ANCA positive.
- MPA (EMA) and MPO-AAV concordance: In total, 80 out of 104 patients (76.9%) classified as MPA by the EMA algorithm were MPO-ANCA positive.
- EGPA Classification: EGPA patients showed a high concordance with ANCA-negative status or MPO-ANCA positivity.

#### ACR/EULAR criteria vs. ANCA specificity

When comparing the 2022 ACR/EULAR criteria with ANCA specificity-based classification (Fig. 3):

- Strong Agreement for MPA/MPO-AAV: Overall, 182 out of 187 patients (97.3%) classified as MPA by the 2022 ACR/EULAR criteria were MPO-ANCA positive.
- Strong Agreement for GPA/PR3-AAV: Overall, 55 out of 71 patients (77.5%) classified as GPA by the 2022 ACR/EULAR criteria were PR3-ANCA positive.
- EGPA classification: Overall, 16 patients classified as EGPA by the 2022 ACR/EULAR criteria were predominantly ANCA-negative or MPO-ANCA positive.

### Statistical analysis of agreement between classification systems

Cohen’s kappa coefficients were calculated to assess the level of agreement between classification methods (Tables 2, 3, and 4).

**Table 2:** Agreement between EMA algorithm and 2022 ACR/EULAR criteria

Classification	Positive (EMA)	Negative (EMA)	Kappa	95% CI
GPA	58	146	0.302 ± 0.047	0.209–0.395
MPA	93	113	0.371 ± 0.044	0.284–0.457
EGPA	16	283	0.725 ± 0.079	0.571–0.880

*MPA* microscopic polyangiitis, *GPA* granulomatosis with polyangiitis, *EGPA* eosinophilic GPA

**Table 3:** Agreement between EMA algorithm and ANCA specificity-based classification

Classification	Positive (EMA)	Negative (EMA)	Kappa	95% CI
GPA/PR3-AAV	42	155	0.226 ± 0.046	0.136–0.317
MPA/MPO-AAV	80	105	0.278 ± 0.040	0.201–0.356
EGPA/ANCA-negative AAV	19	273	0.647 ± 0.077	0.497–0.798

*AAV* ANCA-associated vasculitis, *ANCA* antineutrophil cytoplasmic antibody, *MPA* microscopic polyangiitis, *GPA* granulomatosis with polyangiitis, *EGPA* eosinophilic GPA, *MPO* myeloperoxidase, *PR3* proteinase 3

**Table 4:** Agreement between 2022 ACR/EULAR criteria and ANCA specificity-based classification

Classification	Positive (ACR/EULAR)	Negative (ACR/EULAR)	Kappa	95% CI
GPA/PR3-AAV	55	217	0.663 ± 0.050	0.566–0.762
MPA/MPO-AAV	182	100	0.806 ± 0.035	0.738–0.874
EGPA/ANCA-Negative AAV	16	219	0.221 ± 0.052	0.119–0.322

*AAV* ANCA-associated vasculitis, *ANCA* antineutrophil cytoplasmic antibody, *MPA* microscopic polyangiitis, *GPA* granulomatosis with polyangiitis, *EGPA* eosinophilic GPA, *MPO* myeloperoxidase, *PR3* proteinase 3

#### EMA algorithm vs. 2022 ACR/EULAR criteria

- GPA classification agreement: kappa = 0.302 (95% CI, 0.209–0.395), indicating fair agreement.
- MPA classification agreement: kappa = 0.371 (95% CI, 0.284–0.457), indicating fair agreement.
- EGPA classification agreement: kappa = 0.725 (95% CI, 0.571–0.880), indicating substantial agreement.

#### EMA algorithm vs. ANCA specificity-based classification

- GPA/PR3-AAV agreement: kappa = 0.226 (95% CI, 0.136–0.317), indicating fair agreement.
- MPA/MPO-AAV agreement: kappa = 0.278 (95% CI, 0.201–0.356), indicating fair agreement.
- EGPA/ANCA-negative AAV agreement: kappa = 0.647 (95% CI, 0.497–0.798), indicating substantial agreement.

#### ACR/EULAR criteria vs. ANCA specificity-based classification

- GPA/PR3-AAV agreement: kappa = 0.663 (95% CI, 0.566–0.762), indicating substantial agreement.
- MPA/MPO-AAV agreement: kappa = 0.806 (95% CI, 0.738–0.874), indicating almost perfect agreement.
- EGPA/ANCA-negative AAV agreement: kappa = 0.221 (95% CI, 0.119–0.322), indicating fair agreement.

#### Summary of kappa agreement (Table 5)

**Table 5:** Summary of kappa agreement between different classification criteria for GPA and MPA

Comparison	GPA kappa (95% CI)	MPA kappa (95% CI)
EMA vs. ACR/EULAR criteria	0.302 (0.209–0.395)	0.371 (0.284–0.457)
EMA vs. ANCA specificity	0.226 (0.136–0.317)	0.278 (0.201–0.356)
ACR/EULAR criteria vs. ANCA specificity	0.663 (0.566–0.762)	0.806 (0.738–0.874)

Kappa values indicate the level of agreement: values between 0.61 and 0.80 represent substantial agreement, and values between 0.81 and 1.00 represent almost perfect agreement

ANCA antineutrophil cytoplasmic antibody, MPA microscopic polyangiitis, GPA granulomatosis with polyangiitis

- The highest agreement was observed between the 2022 ACR/EULAR criteria and the ANCA specificity-based classification for MPA/MPO-AAV (kappa = 0.806).
- There was substantial agreement between the 2022 ACR/EULAR criteria and the ANCA specificity-based classification for GPA/PR3-AAV (kappa = 0.663).
- The EMA algorithm showed lower agreement levels with both the 2022 ACR/EULAR criteria and the ANCA specificity-based classification.

### Key findings

- ANCA specificity enhances classification accuracy: The use of the ANCA serotype as a primary classification criterion demonstrated substantial to almost perfect agreement with the 2022 ACR/EULAR criteria for MPA and GPA.
- Consistency in EGPA classification: EGPA classifications remained consistent across the EMA algorithm and the 2022 ACR/EULAR criteria, suggesting that ANCA specificity is less critical for EGPA diagnosis.
- Reclassification impact: A significant number of patients initially classified as GPA by the EMA algorithm were reclassified as MPA under the 2022 ACR/EULAR criteria and ANCA specificity-based classification, highlighting the impact of updated criteria and the importance of ANCA specificity.

## Discussion

In this retrospective cohort study involving 310 patients with antineutrophil cytoplasmic antibody-associated vasculitis (AAV), we evaluated the utility of using ANCA specificity as a primary criterion for classifying AAV subtypes. Our findings suggest that ANCA serotype-based classification demonstrates substantial to almost perfect agreement with the 2022 ACR/EULAR criteria for microscopic polyangiitis (MPA) and granulomatosis with polyangiitis (GPA). Specifically, the kappa coefficients for MPA/MPO-AAV and GPA/PR3-AAV were 0.806 and 0.663, respectively, indicating strong concordance. These results imply that ANCA specificity may serve as a useful tool in simplifying the classification process for certain AAV subtypes.

The substantial agreement observed between ANCA specificity-based classification and the 2022 ACR/EULAR criteria underscores the potential of the ANCA serotype as an adjunct in the diagnostic evaluation of AAV. The 2022 ACR/EULAR criteria represent a comprehensive framework that integrates clinical, serological, and imaging findings to enhance diagnostic accuracy [3–5]. Our study suggests that ANCA specificity, particularly MPO-ANCA and PR3-ANCA positivity, aligns closely with these criteria for MPA and GPA, respectively.

This alignment may be attributed to the pathophysiological differences between MPO-AAV and PR3-AAV, which manifest in distinct clinical phenotypes. MPO-ANCA is commonly associated with MPA, characterized by renal involvement and pulmonary manifestations such as interstitial lung disease [7]. In contrast, PR3-ANCA is more frequently linked to GPA, often presenting with upper and lower respiratory tract involvement and granulomatous inflammation [8]. By reflecting these underlying differences, ANCA specificity can enhance the precision of subtype classification.

Incorporating ANCA specificity into the classification process may offer practical benefits in clinical settings. A simplified classification approach could facilitate earlier diagnosis and prompt initiation of appropriate therapy, which is crucial given the potential for rapid disease progression and organ damage in AAV [15]. Moreover, the ANCA serotype has been associated with differences in disease course and response to treatment, suggesting that an ANCA-based classification might inform prognostic assessments and therapeutic decisions [16]. Beyond facilitating early intervention, distinguishing MPO-ANCA from PR3-ANCA could prove valuable for long-term monitoring; while PR3-AAV may exhibit higher relapse rates, MPO-AAV often carries a greater risk of chronic organ damage [17–19]. Besides, its strong correlation with established criteria, an ANCA-specific classification approach may streamline clinical workflows, reduce the risk of misclassification, facilitate more precise therapeutic interventions, and improve outcomes. However, prospective validation is needed to confirm if these efficiencies ultimately yield better patient outcomes, including lower morbidity and reduced relapse rates.

An important observation in our study was the reclassification of numerous “GPA” cases (previously defined by the older EMA algorithm) to MPA under the 2022 ACR/EULAR criteria or ANCA-specific classification. This shift suggests that earlier approaches, which relied heavily on clinical and histopathological parameters, may have underemphasized the pivotal role of ANCA serotypes in differentiating these subtypes. Accumulating evidence also links ANCA serotypes to distinct disease trajectories and treatment responses, implying that ANCA-based classification may guide prognostic assessments and refine therapeutic decisions [16]. Notably, employing a serology-first approach can help clinicians promptly distinguish MPA from GPA, leading to more targeted immunosuppression and improved outcomes.

However, it is important to recognize that ANCA testing should complement, rather than replace, comprehensive clinical evaluation. The classification of AAV involves a complex interplay of clinical features, laboratory findings, and histopathological evidence. While ANCA specificity can enhance classification accuracy for certain subtypes, reliance on serology alone may overlook atypical presentations or ANCA-negative cases.

Our study found that the classification of eosinophilic granulomatosis with polyangiitis (EGPA) remained consistent across the EMA algorithm and the 2022 ACR/EULAR criteria, with a kappa coefficient of 0.725, indicating substantial agreement. This suggests that ANCA specificity is less critical for EGPA classification, likely due to its distinct clinical features such as asthma and eosinophilia [12]. Therefore, while ANCA testing is valuable, EGPA diagnosis continues to rely heavily on clinical manifestations. Approximately 40% of EGPA patients are ANCA-positive, and when ANCAs are present, they are more commonly MPO-ANCA [20]. The utility of ANCA testing in EGPA may be more prognostic than diagnostic, certain studies suggest that ANCA-positive EGPA patients are more prone to renal involvement but may have less cardiac involvement than ANCA-negative counterparts, though data remain inconclusive [10, 12]. Data remain limited regarding how this seropositivity affects treatment responses [21]. Additional researches are warranted on treatment responses stratified by ANCA status in EGPA.

Our findings are consistent with prior research suggesting the utility of ANCA specificity in distinguishing AAV subtypes. Hilhorst et al. [7] reported that the ANCA serotype correlates with clinical phenotype and outcomes, supporting its role in classification. Similarly, Cornec et al. [22] highlighted the potential of ANCA specificity to enhance diagnostic precision when integrated with clinical assessment.

However, some studies have emphasized the limitations of ANCA-based classification, particularly due to variability in ANCA testing and the existence of ANCA-negative cases [23–25]. Therefore, while our study supports the utility of ANCA specificity, it also underscores the importance of a comprehensive diagnostic approach. ANCA-negative vasculitis poses an ongoing dilemma. These patients often present with features overlapping those of MPA or GPA yet lack the serologic “signal” crucial for straightforward classification. Hence, our study highlights the need for supplementary diagnostic methods—such as PET-CT imaging or advanced biomarker assays (e.g., immunoglobulin profiling, complement pathway analysis)—to resolve uncertainties and enhance the diagnostic accuracy in ANCA-negative AAV cases.

The limitations of this study include, firstly, the retrospective design may introduce selection bias, and the reliance on existing medical records could result in incomplete data capture. Secondly, the study was conducted across three tertiary care centers, which may limit the generalizability of the findings to other settings, particularly primary care or community hospitals. Finally, ANCA testing methods and interpretation can vary between laboratories, potentially affecting the consistency of results.

Additionally, the study did not assess long-term patient outcomes in relation to the classification systems used. Future prospective studies are needed to evaluate how ANCA specificity-based classification impacts clinical management and patient prognosis over time. Further research should focus on validating these findings in larger, multicenter cohorts with diverse populations to ensure their generalizability. Moreover, exploring the integration of other biomarkers and imaging modalities may enhance the classification and management of AAV.

In summary, our study suggests that ANCA specificity may serve as a valuable adjunct in the classification of AAV, particularly for MPA and GPA. While ANCA serotype-based classification shows substantial agreement with the 2022 ACR/EULAR criteria, it should be used in conjunction with comprehensive clinical assessment. For EGPA, traditional classification criteria remain effective, highlighting the need for a nuanced approach that considers both serological and clinical factors. Our findings contribute to the ongoing discussion on optimizing AAV classification to improve diagnostic efficiency and patient care.

## Data Availability

The datasets analyzed for this study are available from the corresponding author Dr. Shengguang Li (lishengguang@vip.sina.com) upon reasonable request.
